# Opposite trends in incidence of breast cancer in young and old female cohorts in Hungary and the impact of the Covid-19 pandemic: a nationwide study between 2011–2020

**DOI:** 10.3389/fonc.2023.1182170

**Published:** 2023-09-18

**Authors:** Zoltán Kiss, Judit Kocsis, Alíz Nikolényi, Zsolt Horváth, Kata Knollmajer, Angéla Benedek, Máté Várnai, Zoltán Polányi, Krisztina Andrea Kovács, Andrea Berta, István Köveskuti, Eugenia Karamousouli, Tamás Géza Szabó, György Rokszin, Ibolya Fábián, Renáta Bartókné Tamás, Orsolya Surján, Diána Fürtős, György Surján, István Kenessey, András Weber, Zsófia Barcza, Tamás Berki, Zoltán Vokó, Csaba Dózsa, Magdolna Dank, Katalin Boér

**Affiliations:** ^1^ MSD Pharma Hungary Ltd, Budapest, Hungary; ^2^ Department of Oncology, Bács-Kiskun County Teaching Hospital, Kecskemét, Hungary; ^3^ Department of Oncotherapy, University of Szeged, Szeged, Hungary; ^4^ RxTarget Ltd., Szolnok, Hungary; ^5^ University of Veterinary Medicine Budapest, Department of Biostatistics, Budapest, Hungary; ^6^ Department of Deputy Chief Medical Officer II., National Public Health Center, Budapest, Hungary; ^7^ Institute of Digital Health Sciences, Semmelweis University, Budapest, Hungary; ^8^ National Institute of Oncology, National Tumorbiology Laboratory project (NLP-17), Budapest, Hungary; ^9^ Department of Pathology, Forensic and Insurance Medicine, Semmelweis University, Budapest, Hungary; ^10^ Cancer Surveillance Branch, International Agency for Research on Cancer, Lyon, France; ^11^ Syntesia Medical Communications Ltd, Budapest, Hungary; ^12^ Center for Health Technology Assessment, Semmelweis University, Budapest, Hungary; ^13^ Department of Theoretical Health Sciences, University of Miskolc Faculty of Health Sciences, Miskolc, Hungary; ^14^ Cancer Center, Semmelweis University, Budapest, Hungary; ^15^ Department of Clinical Oncology, St. Margaret Hospital, Budapest, Hungary

**Keywords:** breast cancer, incidence, Covid-19, mortality rate, pandemic (COVID19)

## Abstract

**Background:**

This nationwide study examined breast cancer (BC) incidence and mortality rates in Hungary between 2011–2019, and the impact of the Covid-19 pandemic on the incidence and mortality rates in 2020 using the databases of the National Health Insurance Fund (NHIF) and Central Statistical Office (CSO) of Hungary.

**Methods:**

Our nationwide, retrospective study included patients who were newly diagnosed with breast cancer (International Codes of Diseases ICD)-10 C50) between Jan 1, 2011 and Dec 31, 2020. Age-standardized incidence and mortality rates (ASRs) were calculated using European Standard Populations (ESP).

**Results:**

7,729 to 8,233 new breast cancer cases were recorded in the NHIF database annually, and 3,550 to 4,909 all-cause deaths occurred within BC population per year during 2011-2019 period, while 2,096 to 2,223 breast cancer cause-specific death was recorded (CSO). Age-standardized incidence rates varied between 116.73 and 106.16/100,000 PYs, showing a mean annual change of -0.7% (95% CI: -1.21%–0.16%) and a total change of -5.41% (95% CI: -9.24 to -1.32). Age-standardized mortality rates varied between 26.65–24.97/100,000 PYs (mean annual change: -0.58%; 95% CI: -1.31–0.27%; p=0.101; total change: -5.98%; 95% CI: -13.36–2.66). Age-specific incidence rates significantly decreased between 2011 and 2019 in women aged 50–59, 60–69, 80–89, and ≥90 years (-8.22%, -14.28%, -9.14%, and -36.22%, respectively), while it increased in young females by 30.02% (95%CI 17,01%- 51,97%) during the same period. From 2019 to 2020 (in first COVID-19 pandemic year), breast cancer incidence nominally decreased by 12% (incidence rate ratio [RR]: 0.88; 95% CI: 0.69–1.13; 2020 vs. 2019), all-cause mortality nominally increased by 6% (RR: 1.06; 95% CI: 0.79–1.43) among breast cancer patients, and cause-specific mortality did not change (RR: 1.00; 95%CI: 0.86–1.15).

**Conclusion:**

The incidence of breast cancer significantly decreased in older age groups (≥50 years), oppositely increased among young females between 2011 and 2019, while cause-specific mortality in breast cancer patients showed a non-significant decrease. In 2020, the Covid-19 pandemic resulted in a nominal, but not statistically significant, 12% decrease in breast cancer incidence, with no significant increase in cause-specific breast cancer mortality observed during 2020.

## Introduction

Female breast cancer is the most commonly diagnosed cancer worldwide with an estimated 2.3 million new cases (11.7% of all cancer cases), and one of the leading causes of cancer-related death in women (1, 2). The incidence of breast cancer is largely influenced by participation at mammography screening as well as the prevalence of reproductive, hormonal, and lifestyle risk factors (1). Until the 2000s, the incidence of breast cancer was uniformly increasing in most parts of the world; however, due to the reduction in the use of menopausal hormone replacement therapy (HRT) and the plateauing in screening participation, incidence rates have stabilized or even slightly decreased over the past two decades in certain Wester-European countries. Although age-standardized incidence rates (ASRs) still show a slightly increasing trend in Europe as a whole, in Western Europe, ASRs decreased from 126.8 per 100,000 person-years (PYs) in 2012 to 125.5 in 2018 (3, 4). In most of the developing countries, breast cancer mortality rates have increased in parallel with the increasing incidence, however; developed countries have mostly reported decreasing mortality trends during the past two decades. In Europe, the ASR of mortality decreased from 26.0 per 100,000 PYs in 2006 to 21.8 in 2018 (3–5). The reduction of breast cancer mortality could mainly be attributed to early detection by screening programs, to more effective therapeutic modalities, and the decrease in HRT use (6).

The Covid-19 pandemic required the reconfiguration of health service provision and had a serious impact on the capacity of healthcare systems all around the world. As the top priority was to handle the repeated waves of the pandemic, oncological care including cancer screening and diagnostic procedures suffered severe setbacks. Screening programs were suspended in many European countries, which led to delays in diagnosis and significant decreases in the number of newly diagnosed cases (7, 8). Understanding the magnitude of the problem is crucial to develop strategies for addressing this unmet need generated by the pandemic and avoid further increases in preventable cancer deaths.

Global estimates for breast cancer incidence in Hungary have largely been based on mortality data, which cannot be considered an accurate and appropriate basis for evaluation. The Hungarian CONCORD Multiple Cancer Epidemiology study was recently launched to assess the incidence and mortality of the 20 most common cancer types in Hungary between 2011 and 2021 (future extension until 2025), and to evaluate the impact of the Covid-19 pandemic by analyzing differences in monthly incidence and mortality rates in 2020–2021 vs. 2015–2019. The current study was performed as part of the CONCORD program to explore the epidemiology of breast cancer in Hungary between 2011 and 2020, identify potential trends in age-standardized incidence and mortality, and evaluate the impact of the Covid-19 pandemic on breast cancer care in 2020.

## Materials and methods

### Data sources

Our nationwide, retrospective survey was based on the databases of Hungarian National Health Insurance Fund (NHIF) and the Central Statistical Office (CSO). The NHIF database covers almost the entire Hungarian population and contains information on prescription claims, in- and outpatient visits and medical procedures, as well as medical information using ICD-10 codes according to the 10th revision of International Statistical Classification of Diseases and Related Health Problems (ICD-10) (9). The CSO database provides data on age- and sex-specific mortality from all Hungarian citizens annually.

Our study included breast cancer patients (ICD-10 code: C50) diagnosed between January 1, 2011 and December 31, 2020, who had at least 2 occurrences of C50 code in the NHIF database. Patients who died within 60 days of the first C50 code record were also included. A reference period was set from 2009 to 2010 to accurately identify newly diagnosed breast cancer patients and exclude patients with prevalent breast cancer at the start of the time window who had a prior breast cancer diagnosis code. Hungarian population sizes for incidence and prevalence calculations by age and sex, as well as dates and numbers of cause-specific mortality among breast cancer patients were obtained from the Hungarian CSO (10). All-cause mortality data were retrieved from the NHIF database for the cumulative prevalent breast cancer population. Thus, we were able to calculate all-cause mortality as well as cause-specific mortality data for the breast cancer population on a yearly basis.

For the calculation of incidence rates, annual numbers of patients newly diagnosed with breast cancer was given as crude numbers (n); new cases were counted for each calendar year, (between January 1 and December 31). Annual incidence rates are expressed as standardized rates (per 100,000 PYs). In addition, we also calculated annual cumulative incidence as percentages (%) of the total population at risk. Total population at risk was determined by subtracting the number of prevalent breast cancer cases known on January 1 of a given year from the total population of the same year based on annual mid-year population estimates from the CSO (prevalent patient population are counted from the beginning of screening period – 1^st^ of Jan 2009).

For prevalence calculations, the number of breast cancer patients was determined using the annual number of patients who were alive on January 1 of the given year. Patients newly diagnosed in the given year were also included in the annual prevalence. Annual prevalence was expressed as crude numbers (n), in addition, we also calculated prevalence rates as percentages (%) of the total population based on annual mid-year population estimates from the CSO. Age-standardized prevalence per 100,000 PYs were also calculated by sex using the cohort weights from European Standard Population (ESP) 2013 (11).

The calculation of cause-specific mortality rates was based on data from the CSO database. We considered the number of patients who died of breast cancer between January 1 and December 31 of a given year as the number of breast cancer cause-specific deaths. Cancer-specific mortality was expressed as crude numbers (n) and standardized rates per 100,000 PYs. We used standardized incidence and cause-specific mortality rates to evaluate trends in incidence and mortality over time. Total changes and annual changes between 2011–2019, 2011–2015, 2015–2019 and 2020 vs. 2019 were presented as percentages (%).

To allow for direct comparisons with recent and earlier publications, incidence and mortality data were adjusted for age using both ESP 1976 and 2013 for standardization (12). Where crude numbers of any parameter were recorded below 10, we indicated “<10” as the NHIF data protection rule does not allow the presentation of case numbers below 10 in a stratum. In these cases, calculations were run on the exact crude numbers.

The study was approved by the National Ethical Committee (IV/298-2/2022/EKU).

### Statistical analysis

Regression models were used to estimate annual trends with 95% confidence intervals (95% CI). As data were not independent, a block-based bootstrap method was used for time series with a fixed block size of 2. Hungarian population sizes were calculated based on mid-year population sizes published by the Hungarian CSO. The size of the at-risk population was determined based on the difference between mid-year population sizes and the number of previously diagnosed breast cancer patients on January 1 each year. Poisson regression was used for the calculations of annual change of incidence and mortality. In the 2011 to 2019 period, the outcome was the number of patients, the offset was the log of the number of patients at risk or the mid-year population, the explanatory *Brewer HR* were the year. When comparing the periods 2011–2015 and 2015–2019, the outcome was the number of patients, the offset was the log of the number of patients at risk or the mid-year population, the two explanatory variables were the number of years since 2011 and the number of years since 2015. All calculations were performed with R version 3.6.1 (05/07/2019) with package boot version 1.3-20.

## Results

### Data on crude incidence numbers

We identified 8,233 and 7,729 newly diagnosed breast cancer cases from the NHIF database in 2011 and 2019, respectively, corresponding to 0.16% and 0.15% of the total Hungarian population at risk (**Table 1**). Corresponding numbers in 2020 were 6,808 and 0.14%, respectively. The number of identified prevalent breast cancer population increased from 71,907 to 103,353 persons during study period, representing 2.04% of the total female population in 2020. The mean age at diagnosis was 63.4 years both in 2011 and 2019 and did not change significantly in 2020 (62.9 years) (**Supplementary Figure 1**; **Supplementary Table 1**).

**Table 1:** Number of incident and prevalent breast cancer cases, and cause-specific and all-cause mortality of breast cancer patients.

	2011		2012		2013		2014		2015		2016		2017		2018		2019		2020	
Incident population																				
Female with new diagnosis (n, % of female population at risk)	8,233	0.16%	7,798	0.15%	7,847	0.15%	8,031	0.16%	7,934	0.16%	8,107	0.16%	7,941	0.16%	7,959	0.16%	7,729	0.15%	6,808	0.14%
Prevalent population																				
Female with diagnosis (n, % of female total population)	71,907	1.37%	76,155	1.46%	80,252	1.55%	84,341	1.63%	88,214	1.71%	91,879	1.79%	95,274	1.86%	98,527	1.93%	101,454	1.99%	103,353	2.04%
Cause specific mortality																				
Female died based on CSO (n, % of prevalent population)	2,138	2.97%	2,096	2.75%	2,167	2.70%	2,107	2.50%	2,220	2.52%	2,223	2.42%	2,123	2.23%	2,127	2.16%	2,174	2.14%	2,195	2.12%
All-cause mortality																				
Female died based on NHIF (n, % of prevalent population)	3,550	4.94%	3,750	4.92%	3,942	4.91%	4,061	4.81%	4,442	5.04%	4,546	4.95%	4,706	4.94%	4,802	4.87%	4,909	4.84%	5,274	5.10%

CSO, Central Statistical Office; NHIF, National Health Insurance Fund.

The annual number of breast cancer patients who died from any cause increased from 3,550 in 2011 to 5,274 in 2020, while breast cancer cause-specific mortality varied between 2,096 and 2,223 per year during the study period. The mean age at the time of all-cause death increased from 71.8 (SD ± 13.19) to 75.8 (SD ± 12,03) years between 2011 and 2020 (**Supplementary Figure 1**; **Supplementary Table 1**).

### Age-standardized incidence and mortality rates

**Figure 1:**
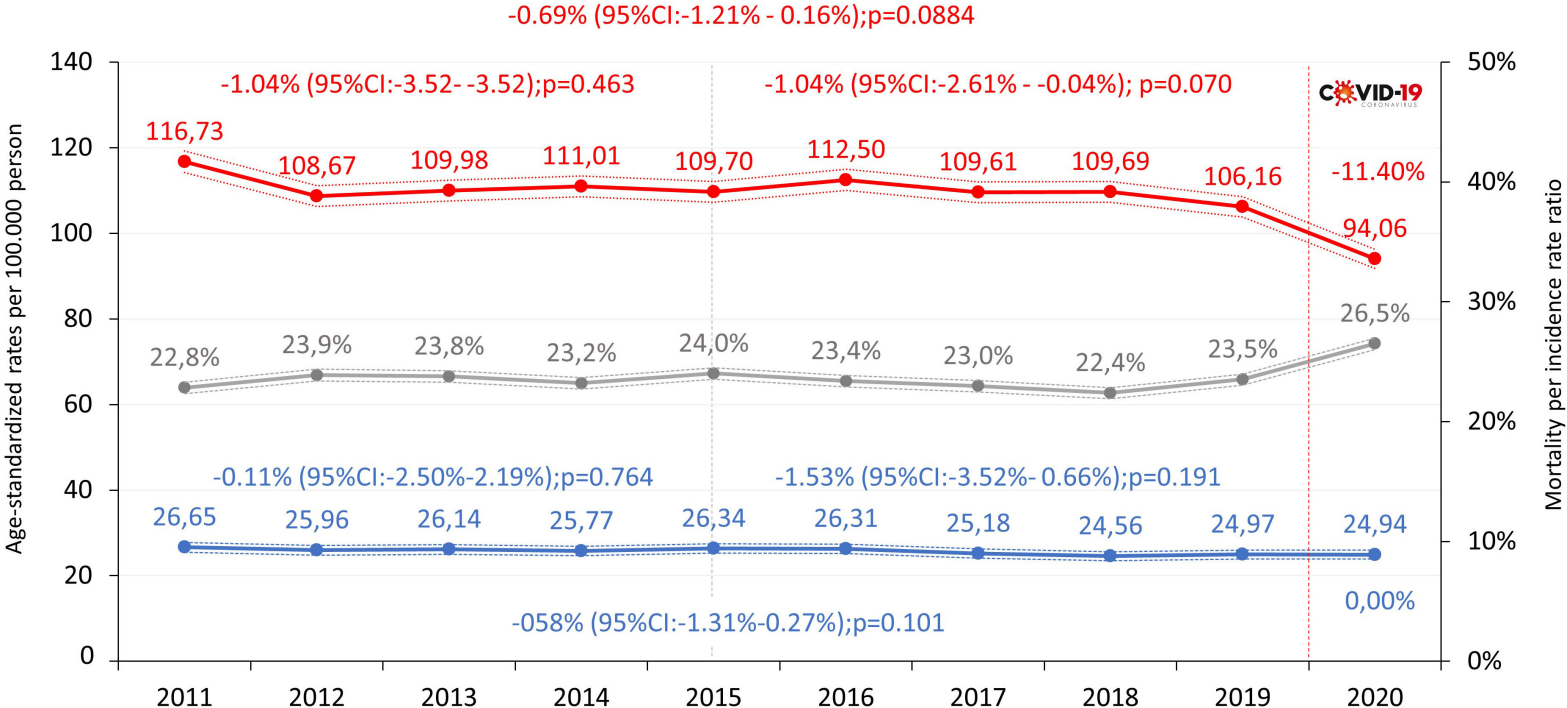
Age-standardized incidence rates (red), mortality rates (blue, ESP 1976) and mortality-per-incidence rate ratio (grey) of breast cancer (C50) in females in Hungary between 2011 and 2020 (per 100,000 person-years; dotted lines represent 95% CI). CI, confidence interval; ESP, European Standard Population.

Age-standardized incidence rates in Hungarian females (ESP 1976) were 116.73 per 100,000 PYs in 2011 and 106.16 per 100,000 PYs in 2019. The mean annual change between 2011 and 2019 was -0.69% (95% CI: -1.21%–0.16%; p=0.09), which was not significant during this period, nor during 2011–2015 or 2015–2019 (**Figure 1**; **Supplementary Table 2**). In 2020, the age-standardized incidence rate decreased by 11.40% compared to 2019. Age-standardized cause-specific mortality rates were 26.65 per 100,000 PYs in 2011 and 24.97 per 100,000 PYs among BC patients in 2019, corresponding to a non-significant mean annual decrease of -0.58% (95% CI: -1.31–0.27%; p=0.1). Mean annual changes were not significant in either examined period. Cause-specific mortality rates did not change significantly between 2019 and 2020 (24.97 and 24.94 per 100,000 PYs, respectively). The mortality-per-incidence rate ratio varied between 22.8% and 23.5% in 2011 and 2019, respectively, and increased to 26.5% in 2020 during the first year of the Covid-19 pandemic, because of the decrease of the incidence.

### Age-specific incidence and mortality rates between 2011 and 2019

**Figure 2:**
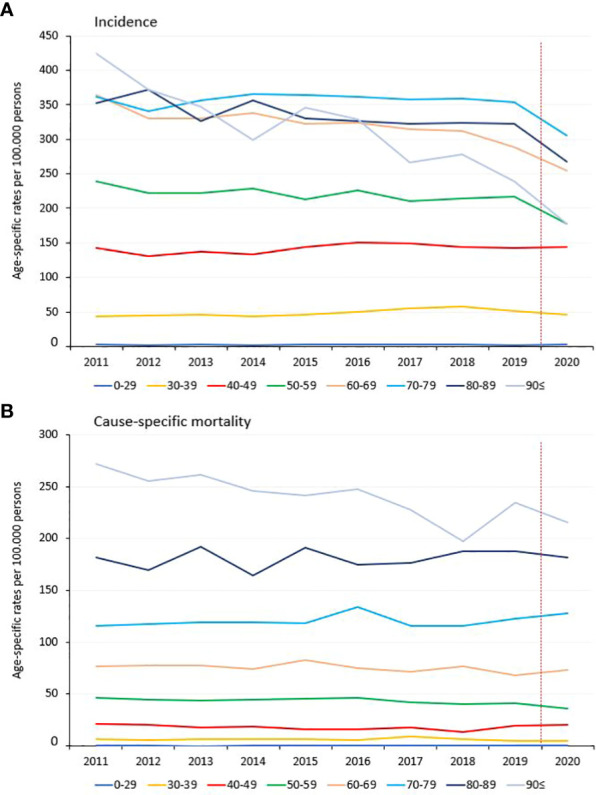
Age-specific incidence **(A)** and mortality **(B)** rates of breast cancer patients (C50) in Hungary between 2011 and 2020 (per 100,000 PYs).

At the beginning of study period, the highest age-specific incidence rate of breast cancer was found in the ≥60 years age cohorts, with 300 to 400 breast cancer patients per 100,000 PYs. In the 50–59 years, 60–69 years, 80–89 years, and ≥90 years age cohorts, age-specific incidence rates significantly decreased between 2011 and 2019 (-8.22%, -14.28%, -9.14%, and -36.22%, respectively), while there was a significant, 32.09% increase in the age cohort of 30–39 years (**Figure 2A**; **Supplementary Table 2**). In the 0-49 aggregated cohort, the increase was 30.02% while there was a 5.97% decrease in the older cohort (50 and older). As expected, age-dependent cause-specific mortality rates increased by age, but we did not find any significant change in any of the age cohorts between 2011 and 2019, neither in the aligned age 0-49 and age 50 and over population (**Figure 2B**; **Supplementary Table 3**).

### Incidence and mortality rates in 2020 vs. 2019 – impact of the Covid-19 pandemic

**Figure 3:**
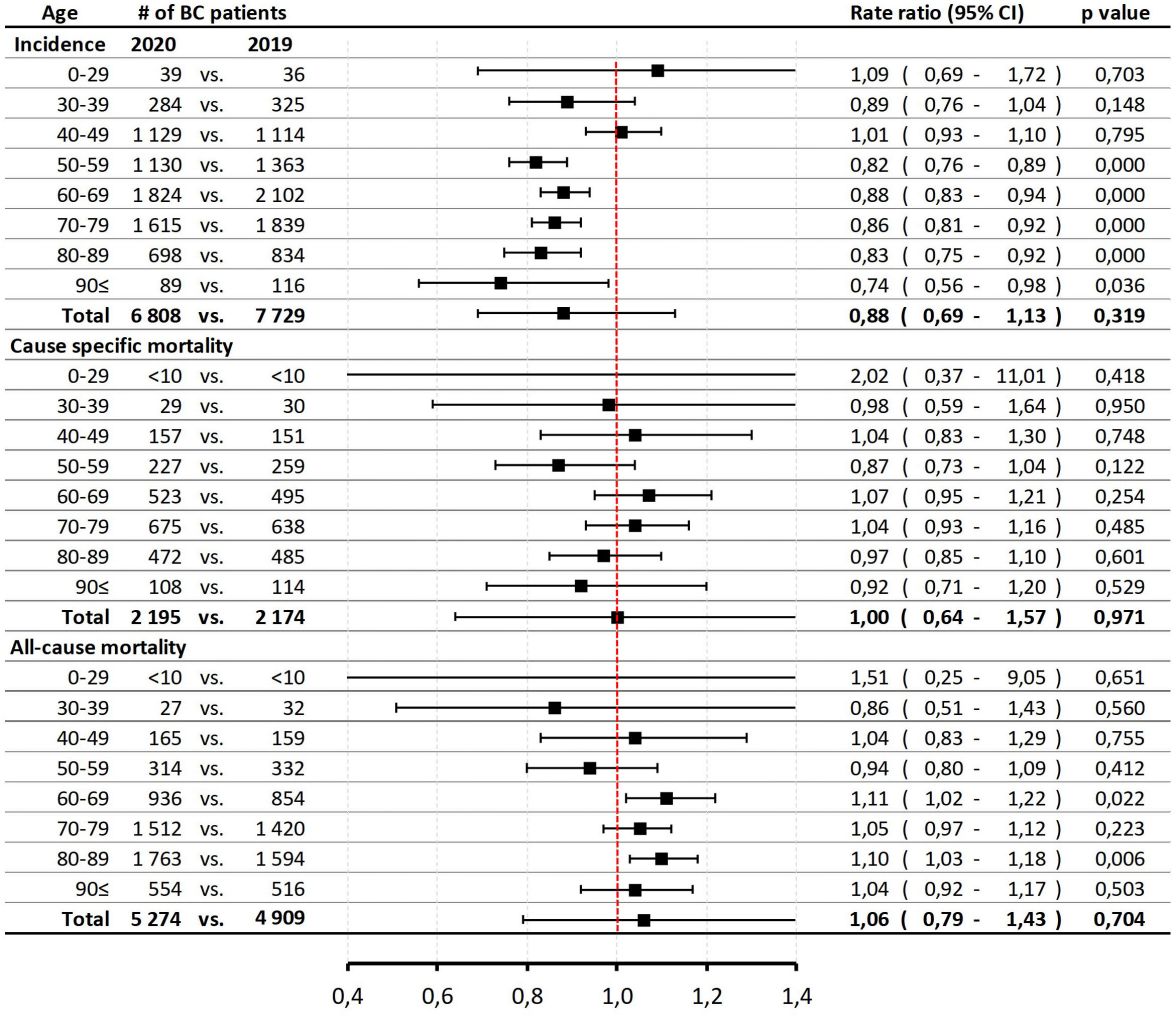
Age-specific incidence and mortality rates (cause-specific and all-cause mortality) of breast cancer patients (C50) in 2020 vs. 2019 due to the impact of Covid-19 pandemic restrictions. BC, breast cancer; CI, confidence interval.

The incidence of breast cancer decreased non-significantly from 2019 to 2020; with an incidence rate ratio of 0.88 (95% CI: 0.69–1.13; p=0.3192; 2020 vs. 2019). There were more noticeable and significant decreases in older age cohorts, with -26% in the ≥90 years and -12–18% in the 60–69 and 50–59 years age cohorts. However, we found no significant decreases in younger age groups (**Figure 3**). Cause-specific mortality rate ratios did not show any significant changes in any age cohorts or in the total population (RR: 1.00; 95% CI 0.86–1.15; p=0.97) in 2020 vs. 2019. The all-cause mortality rate increased by 6% in 2020 compared to the preceding year, although the change was not significant (RR: 1.06; 95% CI: 0.79–1.43; p=0.7). In the 80–89 years and 60–69 years age cohorts, all-cause mortality rates significantly increased by 10 and 11%, respectively.

### Incidence and mortality rates in Hungary compared to other European countries

**Figure 4:**
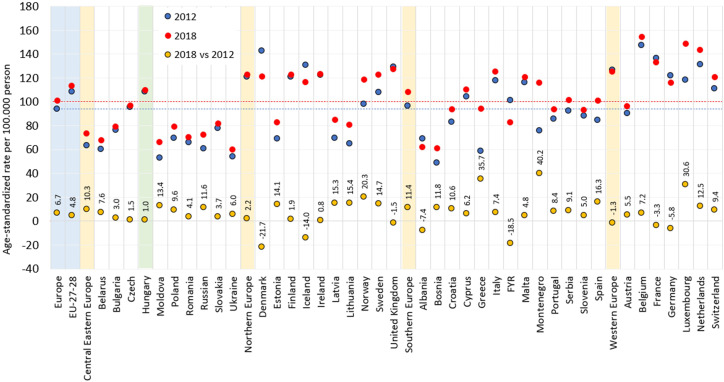
Age-standardized incidence rates of female breast cancer per 100,000 PYs in European countries and Hungary based on the NHIF survey in 2012 and 2018 (ESP 1976) and change in incidence rates between 2012 and 2018. ESP, European Standard Population; NHIF, National Health Insurance Fund; PYs, person-years. Description: EU-27-28: 27 and 28 European Union member states in 2012 and 2018.

In our current study, the age-standardized incidence of breast cancer was 108.7 per 100,000 PYs in 2012 and 109.7 per 100,000 PYs in 2018, which were higher than European average (94.2 and 100.9, respectively) but similar to EU-27 countries’ average (108.8 and 113.6, respectively) based on Ferlay’s reports. Hungarian age-standardized incidence rates were by far the highest in the Central Eastern European region, with 63.4 and 73.7 per 100,000 PYs in 2012 and 2018, respectively (**Figure 4**). On the other hand, the difference between incidence rates in 2018 vs. 2012 was 1% in Hungary, the lowest in the Central Eastern European region and similar to Western and Northern Europe.

**Figure 5:**
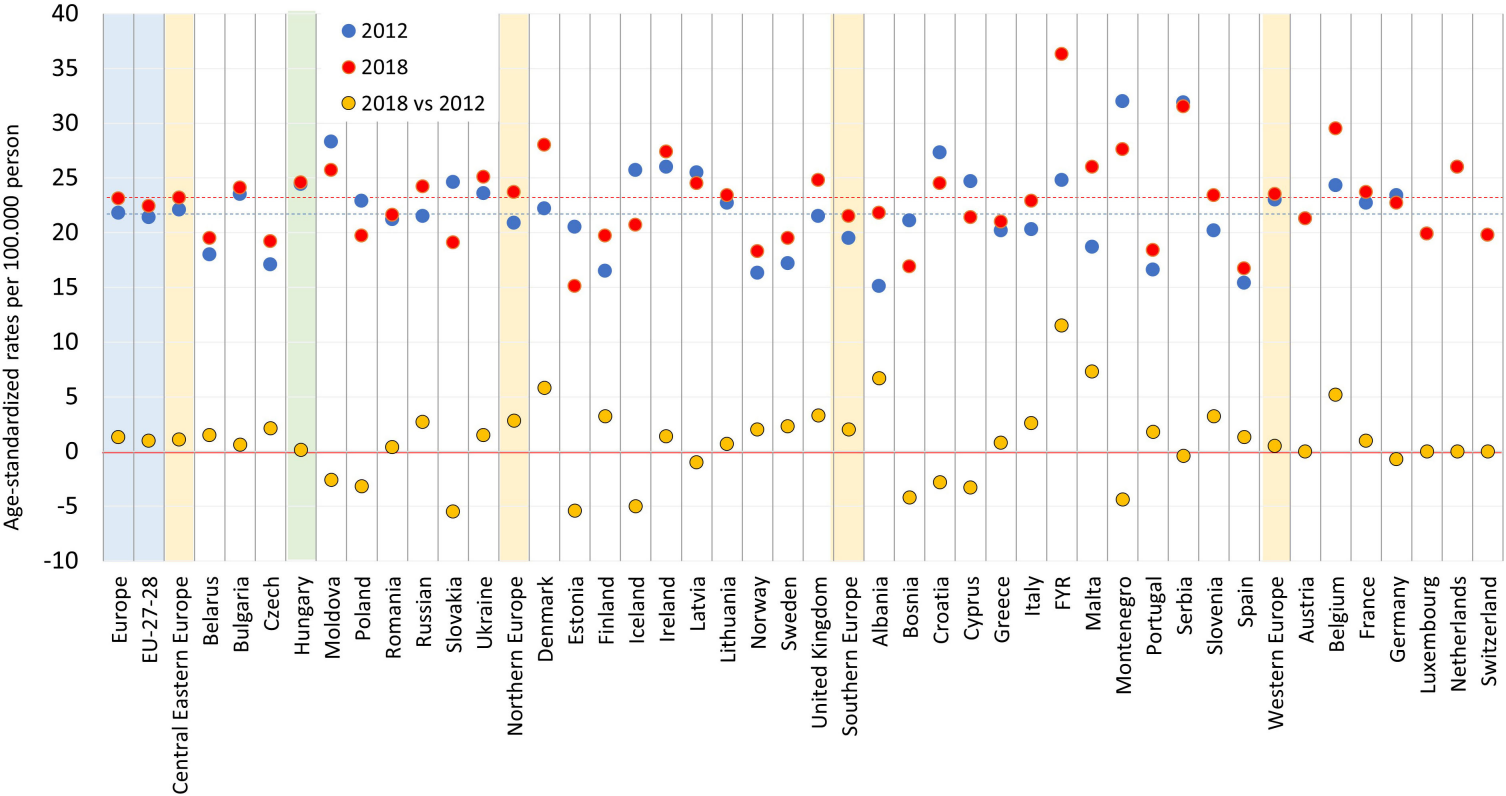
Age-standardized mortality rates of female breast cancer (female) per 100,000 PYs in European countries and Hungary based on the CSO database in 2012 and 2018 (ESP 1976) and change in mortality rates between 2012 and 2018. ESP: European Standard Population; CSO: Central Statistical Office; PYs: person-years. Description: EU-27-28: 27 and 28 European Union member states in 2012 and 2018.

The age-standardized mortality rate of breast cancer was 24.42 per 100,000 PYs in 2012 and 24.58 in 2018, which was slightly higher than the European (21.8 and 23.1) and EU-27 average (21.8 and 23.1). In addition, mortality rates were also among the highest in Central Eastern Europe, where average rates were 22.1 and 23.2 in 2012 and 2018, respectively (**Figure 5**). There were no relevant changes in age-standardized mortality rates between 2012 and 2018 in Hungary or in the Central Eastern European region.

## Discussion

This nationwide, retrospective study was performed as part of the Hungarian CONCORD Multiple Cancer Epidemiology program to assess incidence and mortality rates of breast cancer in Hungary between 2011 and 2019. We also examined the impact of the Covid-19 pandemic on the epidemiology of breast cancer in 2020. Slight, non-significant annual decreases in age-standardized breast cancer incidence have been found between 2011 and 2019, with significant decreases in older age groups, while oppositely, a significant increase was observed in incidence rate among young females. We could not detect any significant decrease in cause-specific breast cancer mortality during the study period in any of the age cohorts, nor in the aligned age cohorts. In the Covid-19 pandemic period a non-statistically significant 12% decrease in newly diagnosed breast cancer cases could be seen, but there were no significant changes in mortality rates yet till the end of 2020, nevertheless, we may expect increase later during the Covid-19 pandemic.

In recent years breast cancer rates have risen with significant variations among countries and regions (13). In certain parts of the world such as China and South Korea, incidence and mortality rates have rapidly increased during the past two decades. This phenomenon could be attributed to epidemiological and demographic changes as well as the increasing prevalence of main risk factors.

On the other hand, the extensive use of effective screening programs and the significant reduction in the use of hormone-replacement therapy (HRT) have led to a decrease in breast cancer incidence in a number of developed countries (14, 15). HRT gained popularity during the 1990s primarily in developing countries, and was accompanied by a parallel increase in breast cancer incidence rates in these regions (13, 16). Finally, a randomized trial conducted by the Women Health Initiative demonstrated the association between HRT and the significantly increased risk of breast cancer, heart disease, stroke, and thromboembolic events in the early 2000s (17). The change in the use of HRT resulted in a change of trend in breast cancer incidence and mortality in developed countries, especially in women aged ≥50 years. The association between the decline in use of HRT and a decrease in breast cancer incidence has been showed by several studies. In Australia, a substantial, 40% reduction in HRT prescriptions from 2001 to 2003 resulted in a significant, 6.7% decrease in the incidence of invasive breast cancer in women aged ≥50 years (18). Similarly, a study conducted in Canada found a 9.6% decline in breast cancer incidence rates from 2002 to 2004 among women aged 50–69 years, which coincided with a significant, 7.8% drop in the use of HRT (19). In Europe, decreases in breast cancer mortality were reported in Germany, France, United Kingdom, Finland, and Denmark between 2012 and 2018 (3, 4). France was known one of the highest incidence rates of breast cancer in Europe. Incidence rates started to decrease there in the early 2000s, which was attributed to the substantial reduction of the use of menopausal HRT (20, 21). In Norway, the sharp decrease in the use of HRT and the implementation of the Norwegian Breast Cancer Screening Program in 2005 and resulted in a decrease in incidence rates of breast cancer in the age group of 50–69 years starting from 2003 (22). Recently, Chen et al. confirmed the detectable decline in breast cancer incidence in Western Europe and North America (23).

Our findings regarding breast cancer incidence trends among older females (≥50) are in line with international observations. In our study, age-standardized breast cancer incidence showed a slight, non-significant mean annual decrease of 0.7% during the study period, with significant decreases in the 50–59 years, 60–69 years, 80–89 years, and ≥90 years age cohorts from 2011 to 2019 (-8.22%, -14.28%, -9.14%, and -36.22%, respectively). Since the participant rate in mammography screening did not change over the past two decades in Hungary (24) it is possible that the observed changes in breast cancer incidence could be attributed to the decrease in the use of HRT, however, we don’t have exact data on the HRT consumption from the previous decades, only on available publication reports that percentage of postmenopausal women using HRT was found to be 2.13% in Hungary, which is lower than Western-European average (25). Nevertheless, Hungarian breast cancer incidence rates can still be considered outstandingly high among Central Eastern European countries. Although incidence rates in Hungary were close to the Western and Northern European average in 2012 and 2018 based on Ferlay’s reports, Central Eastern European countries with the exception of the Czech Republic reported much lower incidence rates in the same years. This may be explained by differences in the effectiveness of screening programs and diagnostic procedures (**Figure 5**) considering that age-standardized mortality rates were comparable in these regions, on the other hand, difference in HRT use within CEE countries could also lead to such result.

Opposite to older cohorts, we found an increase in breast cancer incidence among women under age 50, which is also in line with findings in recent years (26). In a recently published study, average annual percent change in breast cancer incidence was 2.6% in Germany, 1.25% in Austria, 1.91 in Slovakia and 1.62% in Czech Republic (all significant) (23), while it was 3.34% (95%CI: 1.98 – 5.37) in Hungary between 2011 and 2019. Several factors may play a role in the increase of breast cancer incidence among young females. As they are below the age of postmenopausal HRT treatment, this factor could not impact the early incidence of breast cancer. A multi country analyses which was conducted by Collaborative Group on Hormonal Factors in Breast Cancer showed a 24% increased BC risk among current oral contraceptive users, but less among those, who stopped the therapy within 10 years (27). As full-term pregnancy leads to a 3% reduction in premenopausal breast cancer risk (28) and every 12 months of breast-feeding provides a 4.3% risk reduction also in premenopausal BC (29), hence, as the first maternity is prolonged to older ages, this phenomenon could also increase the incidence of early BC among young females. Excess body weight also increases the risk of BC *via* altered steroid metabolism and elevated estrogen level (30) resulting in a 25% higher BC risk (31), especially in case of central obesity, where this excess risk for BC was found to be 39% (32). Prevalence of childhood and young adult obesity has been increasing in recent decades (33, 34), which may play an essential role in increasing trend of BC in premenopausal female cohorts in developing countries. Similar trends were found in Hungary over the previous decades, where the ratio of the overweight and even the number of obese persons increased significantly in younger generation (35), despite that Hungary was already among the countries with highest rate of overweight (36) which may also play essential role in increasing trend of BC among young females. Besides, we must consider, that increasing effectiveness of breast cancer screening in younger females could also increase the number of diagnose BC among the mentioned other factors. Finally, the increasing number of IVF could be considered as a background risk of increasing incidence in BC, whoever, recent meta-analyses on 8 cohort studies (total cohort size of 1,554,332) found no significant association between IVF and breast cancer risk, hence, at present, IVF does not seem to impart increased breast cancer risk (37).

Moreover, family history is a significant risk factor for breast cancer incidence. A recent report from 2017 analyzed breast cancer risk in relation to first-degree family history using a family history score (FHS) (38). The study included a cohort of over 113,000 women from the general UK population and utilized the Generations Study database. The family history score accounted for the expected number of family cases based on the family’s age-structure and national cancer incidence rates. The authors observed a significant increase in breast cancer risk with a greater family history score, ranging from 3.5-fold risk between the lowest and highest FHS groups. While family history may contribute to the rising incidence of breast cancer in young Hungarian females, the available NHIF database did not provide information on family history of BC, limiting our ability to explore this association.

Breast cancer is one of the leading causes of cancer death in women worldwide (4). However, recent studies have reported a steady decrease in mortality rates in developed countries, due to several reasons such as the decrease of incidence, the rapid decrease in the use of HRT, effective screening programs allowing earlier detection and better prognosis, and the availability of innovative treatment options (39–41). A number of clinical trials showed that mammography screening led to a mortality reduction, for instance, in a study conducted in the U.K., participation in breast cancer screening led to a 35% reduction in mortality risk, which demonstrates the clear benefit of screening programs (36). A number of recent analyses have confirmed the favorable trends in breast cancer mortality in European countries and predicted further decreases for the upcoming years (37, 42). In our study, we found a non-significant, -0.58% mean annual decrease in age-standardized breast cancer mortality between 2011 and 2019, with a more pronounced decrease in patients aged 50–69 years. Hungarian age-standardized mortality rates were above the European average (24.58/100,000 PYs vs. 21.8/100,000 PYs, respectively). Several reasons can be attributed to this phenomenon. First, although breast cancer screening programs have been implemented for almost 20 years in Hungary, participation rates were still below 50% in the 2000s, which seems significantly lower than the ideal 70% required to achieve a significant impact on breast cancer mortality (43–45). In addition, a recent Hungarian cross-sectional study revealed significant knowledge gaps in the field of breast cancer screening recommendations among women. Only 35.2% of women and 86.6% of screening attendees knew about the recommended age for first mammography. Only, 33.9% and 12.9% were aware of the recommended screening frequency. Moreover 7.0% and 5.9% had sufficient knowledge about risk factors, and 16.7% and 28.9% had knowledge about the signs and symptoms of breast cancer, respectively (46). Furthermore, Hungary also faces significant challenges in terms of breast cancer risk factors including smoking habits, high fat intake, high BMI, lack of physical exercise, and alcohol consumption (47, 48). Based on a survey carried out in 2010, the proportion of women smoking regularly or occasionally was 31.7% and showed an increasing trend until then (49), which level remained in the same range till the end of this decade (50). Although, another report published in 2013 demonstrated the stabilization of smoking prevalence in Hungarian women (24–25%), Hungary still ranks high among European Union member countries with a smoking rate of 17.3% in females, well above the E.U. average of 14.8% (51, 52). Furthermore, obesity is also a significant concern in Hungary with more than 50% of women being overweight, which is also among the highest in the E.U (53). These data highlight that there is still room for improvement in the field of breast cancer screening, risk factor management, and disease awareness in Hungary.

The Covid-19 outbreak reached Europe on January 24, 2020, when the first case was identified in France (54). A pandemic was soon declared by the World Health Organization (WHO) during the first powerful wave in spring 2020 (55). After a relatively low-intensity summer period of the outbreak, the second wave resulted in more than 83 million confirmed cases and 1.9 million deaths worldwide by the end of the year. Although the first pandemic wave was less powerful in Hungary compared to Western European countries, the second wave from October to December 2020 resulted in relevant excess mortality by the end of 2020 compared to the preceding years (56). During both waves, significant restrictions and lockdown measures were implemented by the government to minimize the impact of the outbreak. Restrictions imposed by various countries had a significant impact on cancer care all across Europe, especially on cancer screening programs. A study from the Netherlands revealed that the first wave of the pandemic resulted in a 50% reduction in the weekly number of newly diagnosed breast cancer cases in the age group of 50–74 years, which then returned to the estimated actual numbers shortly after the end of the lock-down (7). In Slovenia, a two-month lock-down on cervical cancer screening programs resulted in a dramatic decline in screening (-92%), follow-up visits (-70%), HPV testing (-68%), and invasive diagnostic procedures (-47%) (57). A study conducted in the U.K. reported a 63% reduction in the monthly number of suspected colorectal cancer cases, and a 92% decrease in the monthly number of colonoscopies during the first pandemic wave (58). Data from the Netherlands Cancer Registry showed considerable decrease in new cancer diagnoses during the first lock-down compared to the pre-Covid era for all cancer types (59). A recent Hungarian publication also reported a 15.5% decrease (95%CI 2.5% to 27.0%), though during a longer pandemic period, till the second half of 2021 (60). Based on another Hungarian publication, which analyzed both diagnostic and screening mammography examinations, between 2012 and 2019 the coverage (screening and diagnostic mammography) varied between 48.1–51.5 percent, which decreased onto 31.8% in 2020–2021 pandemic period. The yearly number of centrally organized mammography examinations decreased from 219,072 in 2019 by -25.79% onto 162,584 in 2020 which further decreased in 2021 onto 131,182 (61).

In line with these observations, our study showed an 11.58% lower age-standardized breast cancer incidence in 2020 compared to 2019, with the most significant reductions observed in the most vulnerable age groups (women aged ≥50 years). The decrease corresponds to more than 900 female patients who might have been diagnosed in later stages of breast cancer due to the disruption of screening services. Our findings underline the importance of focusing much more on breast cancer screening after the consolidation of the Covid-19 pandemic to prevent long-term increases in breast cancer burden.

We found no significant increase in breast cancer mortality during the first year of the pandemic compared to 2019, which suggests that patients who had been diagnosed prior to or during the Covid-19 pandemic were successfully managed despite the overburdened healthcare system and the significant, 7.7% cumulative excess mortality in 2020 compared to the preceding years (55). It should be noted that no comparative analyses of breast cancer mortality among patients with and without SARS-CoV-2 infection have been performed. In addition, there was no information on the proportion of women diagnosed with SARS-CoV-2 infection among BC patients during the study period. Therefore, it is hard to draw meaningful conclusions regarding the direct impact of coronavirus infection on breast cancer mortality. We can assume that deceased SARS-CoV-2 breast cancer patients were coded as Covid-19 related deaths and may not appear in breast cancer mortality statistics for 2020. Our assumption is supported by the fact that a 6% increase was detected in all-cause mortality among BC patients in 2020 compared to 2019 without an increase in breast cancer cause-specific mortality.

As discussed in previous section, based on our and international findings, a notable reduction in breast cancer incidence was observed during the initial year of the Covid-19 pandemic. However, it is anticipated that during the post-pandemic phase, or even in the latter stages of the pandemic, previous delays in screening, diagnosis, and treatment caused by the Covid-19 pandemic may result in an increase in breast cancer incidence. These effects could lead to a higher proportion of later-stage diagnoses and, in turn, a rise in excess cancer deaths. Additionally, the declining trend in mortality projected for certain cancers could be slowed or even reversed due to these consequences (62). As our analysis was conducted till the end of 2020, we were not able to measure the incidence and mortality trends in post-Covid era. Besides, due to the short post-Covid period, only few reports were available on the incidence change after the Covid-19 pandemic. For example, a report from the English National Health Service Breast Screening Programme showed an 9% lower overall number of referrals in financial years (April–March) of 2020/21 and 9% higher in 2021/22 compared to 2019/20 (63). They also found that in 2019/20 (April–March), the year before the pandemic, the number of first treatments for breast cancer was 49,050, while this number was 23% lower in 2020/21 and already 2% higher in 2021/22. In summary, the authors suggest that there may be ~10,300 “missing” women with breast cancer since the start of the pandemic in England. A Japanese study found significantly higher later stage patients (stage IIB or higher) among diagnosed BC population during the Covid-19 pandemic, besides they estimated a worse, long term survival rate in the pandemic group than in the non-pandemic group (83.9% vs. 87.9%) (64). A recent systematic review of 74 studies found reductions in breast cancer screening volumes and diagnoses, and also reported a higher proportion of advanced-stage cancers at diagnosis (65). However, these authors also suggested that more research is needed to understand the long-term impact of the pandemic on breast cancer care, hence, the epidemiology studies of upcoming years will play a significant role in evaluating the long-term impact of the Covid-19 pandemic on cancer care

Future analyses from 2021–2022 may provide more information on the overall impact of the pandemic on breast cancer care. Modeling studies from England have predicted 148–687 additional breast cancer deaths for the upcoming 10 years due to the suspension of screening services and resulting delays in diagnosis (66), while a U.S. collaborative modeling study projected 2,487 cumulative excess breast cancer deaths by 2030 due to reduced screening, delayed diagnosis, or decreased use of chemotherapy in early stages (67). These findings underline the importance of addressing the substantial cumulative deficit in screening and diagnostic evaluations and devising strategies to resume breast cancer screening, diagnosis, and treatment services.

Our study has certain strengths and limitations. Its strengths are in the robust number of identified breast cancer patients, the carefully analyzed and cleaned data, the 10-year-long follow-up period, the nationwide nature of the NHIF database and linking it to the CSO mortality data. All these factors provide us with a solid foundation for drawing conclusions. Furthermore, the annual numbers of newly diagnosed breast cancer patients were largely consistent with results from the National Cancer Registry, however, there are some differences in the analysis of different age cohorts. The slight differences detected between the two datasets were mainly due to differences in methodology with regards to the identification of patients with certain cancer types. The limitations lay in the fact that NHIF database does not contain any information on the histology of breast cancer, TNM stage, ECOG status of the patients. Therefore, we were not able to assess the incidence of breast cancer by histological and molecular subtypes, nor the impact of patient-related factors on breast cancer mortality.

## Conclusion

We found significant decreases in breast cancer incidence in older age cohorts over the past decade in Hungary, which is in line with incidence trends reported by developed countries. Oppositely, over 30% increase in breast cancer incidence was reported among young, premenopausal females, which may relates mostly to increasing young obesity and the increased age of the first pregnancy in Hungary. There was no detectable decrease in age-standardized breast cancer mortality, which is still higher than the European average. The results underline the importance of improving breast cancer screening practices and managing breast cancer risk factors at national level. Our study also found a nominal 12% decrease in the incidence of breast cancer during the Covid-19 pandemic, which might be attributable to a reduced number of screenings due to patient fear of infection. There was no significant increase in mortality in 2020. Our data are in line with international findings and highlight the significant unmet need in the field of breast cancer management created by the Covid-19 pandemic.

## Data availability statement

The raw data supporting the conclusions of this article will be made available by the authors, without undue reservation.

## Ethics statement

Approval was obtained from the Central Ethical Committee of Hungary (IV/298- 2/2022/EKU). The study is based on anonymized data collected for financial purposes by the NHIF of Hungary, thus it does not include images or any other personal data that may be used to identify any person.

## Author contributions

ZK and LR conceptualization. GR: Conceptualization, Methodology, Writing - Original Draft, MD, KB: Supervision, Writing - Review & Editing. JK, AN, ZH, RBT, OS, DF, GS, IKe, AW, CD: Conceptualization, Validation, Review. KKn, ABen, MV, ZP, KKo, ABer, IKo, EK, TS: Conceptualization, Validation. ZK: Investigation, Conceptualization, Methodology, Writing - Original Draft, Visualization. TB, ZV: Methodology, Supervision, Review. GR: Conceptualization, Validation, Review, Data Curation. IF: Data Curation. ZB: Writing - Review & Editing, validation and writing, MD and KB. All authors contributed to the article and approved the submitted version.

## Supplementary material

Supplementary Figure 1 Mean age of female breast cancer patients at diagnosis and at the time of death (all-cause mortality).
