# Supplementary figures and images for: Opposite trends in incidence of breast cancer in young and old female cohorts in Hungary and the impact of the Covid-19 pandemic: a nationwide study between 2011–2020
Source: Front Oncol. 2023 Sep 18;13:1182170. doi: 10.3389/fonc.2023.1182170 (PMC10545848; doi:10.3389/fonc.2023.1182170)

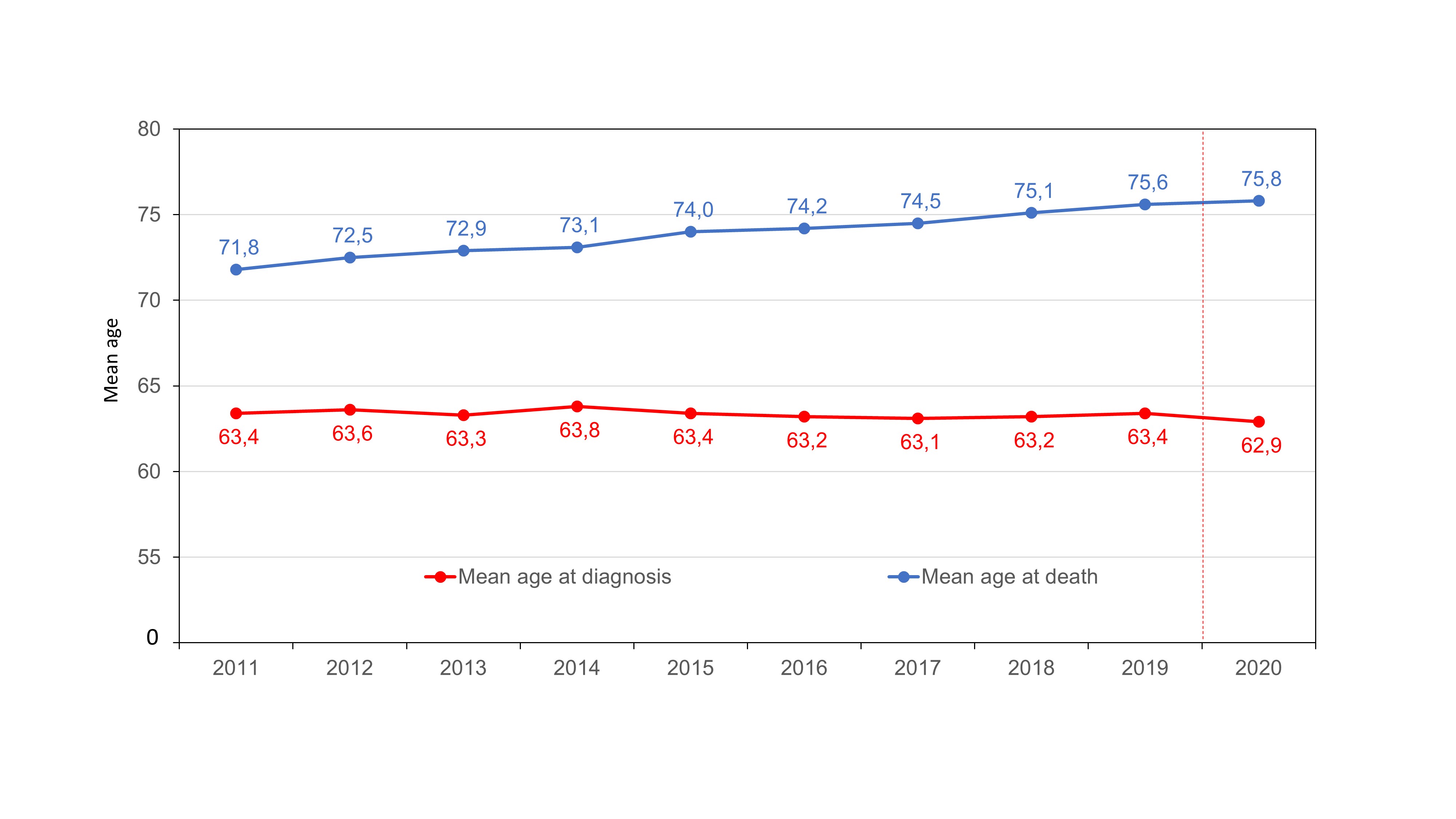

Supplement: Supplementary Figure 1 — Mean age of female breast cancer patients at diagnosis and at the time of death (all-cause mortality). [file Image_1.jpeg]
